# Experimental evaluation of the patient-specific haemodynamics of an aortic dissection model using particle image velocimetry

**DOI:** 10.1016/j.jbiomech.2022.110963

**Published:** 2022-03

**Authors:** Gaia Franzetti, Mirko Bonfanti, Shervanthi Homer-Vanniasinkam, Vanessa Diaz-Zuccarini, Stavroula Balabani

**Affiliations:** a Department of Mechanical Engineering, University College London, London, UK; b Wellcome/EPSRC Centre for Interventional and Surgical Sciences (WEISS), Department of Medical Physics and Biomedical Engineering, University College London, London, UK; c Leeds Teaching Hospitals NHS Trust, Leeds, UK

**Keywords:** Aortic Dissection, Particle Image Velocimetry, Haemodynamics, Patient-specific

## Abstract

Aortic Dissection (AD) is a complex pathology that affects the aorta. Diagnosis, management and treatment remain a challenge as it is a highly patient-specific pathology and there is still a limited understanding of the fluid-mechanics phenomena underlying clinical outcomes. Although *in vitro* models can allow the accurate study of AD flow fields in physical phantoms, they are currently scarce and almost exclusively rely on over simplifying assumptions. In this work, we present the first experimental study of a patient-specific case of AD. An anatomically correct phantom was produced and combined with a state-of-the-art *in vitro* platform, informed by clinical data, employed to accurately reproduce *personalised* conditions. The complex AD haemodynamics reproduced by the platform was characterised by flow rate and pressure acquisitions as well as Particle Image Velocimetry (PIV) derived velocity fields. Clinically relevant haemodynamic indices, that can be correlated with AD prognosis – such as velocity, shear rate, turbulent kinetic energy distributions – were extracted in two regions of interest in the aortic domain. The acquired data highlighted the complex nature of the flow (e.g. recirculation regions, low shear rate in the false lumen) and was in very good agreement with the available clinical data and the CFD results of a study conducted alongside, demonstrating the accuracy of the findings. These results demonstrate that the described platform constitutes a powerful, unique tool to reproduce *in vitro* personalised haemodynamic conditions, which can be used to support the evaluation of surgical procedures, medical devices testing and to validate state-of-the-art numerical models.

## 1 Introduction

Aortic dissection (AD) is a serious vascular condition that occurs when a tear in the aortic wall allows blood to flow within the layers of the vessel, leading to the formation of two separate flow-channels, the true (TL) and the false (FL) lumen, separated by the intimal flap (IF). One or more re-entry tears are usually present in the IF as sites of communication between the two lumina, but in some cases a distal FL outflow tear can be missing. ADs involving the ascending aorta – *Stanford Type-A* – usually require immediate surgical intervention. When the dissection involves only the descending aorta – *Stanford Type-B* – the treatment is variable and highly depends on the specific cases. In case of complications, surgical treatment is the preferred choice. However, the optimal treatment of ‘uncomplicated’ Type-B ADs continues to be debated; it is still challenging and controversial in the medical community (Yuan et al., 2020).

Prediction of adverse outcomes in uncomplicated cases of AD could therefore significantly improve clinical management of this pathology. Currently, outcome predictors are mainly anatomical. However, it has been shown that these parameters are not a reliable determinant of dissection progression or rupture (Van Bogerijen et al., 2014). Furthermore, haemodynamic information, such as flow patterns, pressure, velocity and shear rate distributions are important characteristics for this pathology and have the potential to provide a more comprehensive understanding of the disease (Rudenick et al., 2013, Chung et al., 2000a).

### 1.1 Literature review

The earliest reported experimental works of AD are *in vivo* studies performed on animals with a surgery/drug-induced dissection of the aorta, and were mainly used to investigate surgical treatments for AD (Morales et al., 1998). The main drawbacks included limited access for data acquisition – for practical reasons – cost and ethical concerns. In order to overcome these, AD benchtop experiments – either employing an *ex-vivo* vessel or a synthetic replica – started to be employed and allowed the study of haemodynamic variables through different measurement techniques (e.g. Ultrasound (US) Chung et al., 2000a, magnetic resonance imaging (MRI) Birjiniuk et al., 2017 and, only recently, Particle Image Velocimetry (PIV) Zadrazil et al., 2020).

*Ex vivo* studies – leveraging the use of animal or human biological tissue (Peelukhana et al., 2016, Girish et al., 2016, Faure et al., 2014) – have been performed to investigate the factors that affect dissection progression or prognosis. Nevertheless, the surgery-induced entry tear, and simple geometry, are likely to have a non-negligible impact on the results (Dziodzio et al., 2011).

In contrast, thanks to recent developments in manufacturing techniques, *in vitro* studies can employ artificial phantoms to reproduce the geometry of interest with great accuracy. Such *in vitro* platforms provide an opportunity to conduct well controlled experimental studies, not possible with biological tissue. *In vitro* AD studies are still at an early stage of development and only few works have been reported in the literature in the last two decades.

Parametric studies with idealised artificial phantoms have increased the understanding of the pathology and have highlighted the importance of morphological features on the haemodynamic variables (Chung et al., 2000b, Rudenick et al., 2013, Marconi et al., 2017, Zadrazil et al., 2020) (Table 1). Parametric studies can also be used to assess possible treatment methods and their impact on parameters of interest (e.g. flow patterns, pressures and IF motion) (Chung et al., 2000a).

Moving towards more complex anatomical models, Birjiniuk et al. fabricated an AD silicone phantom based on clinical computed tomography (CT) images (Birjiniuk et al., 2017, Birjiniuk et al., 2020) and demonstrated the importance of employing pulsatile flow as close as possible to the physiological one (Table 1).

However, in order to reproduce the *in vivo* condition of AD and achieve the correct flow and pressure behaviour, accounting for the anatomical features of the vessel is not sufficient; dynamic boundary conditions (BCs) should be employed. Almost all AD experimental models reported in the literature employed either none or simplified BCs. A notable exception is the recent study by Bonfanti et al. (2020), which demonstrated that combining clinical data and numerical simulations it is possible to personalise experimental studies by employing dynamic BCs, and therefore reproduce patient-specific AD haemodynamics in the laboratory.

Due to the complexity and the challenges posed by AD, experimental studies are still at their infancy compared to other vascular pathologies (e.g. aneurysms). Despite the importance of accurate reproduction of both anatomical features and fluid dynamics, no other patient-specific *in vitro* AD study has been reported hitherto.

**Table 1:** Summary of key published *in vitro* studies of Aortic Dissection.

Author	Brief description
Chung et al., 2000b, Chung et al., 2000a	The authors used two idealised phantoms (one rigid and transparent, and one compliant and opaque) both with an aortic arch, abdominal branch vessels and a distal bifurcation, to investigate the effects of anatomical and physiological factors on true lumen collapse

Rudenick et al. (2013)	The authors aimed to assess flow patterns and haemodynamics in flexible models and understand the changes involved in tears of different sizes and locations, with regard to both flows and pressures. More importantly, they demonstrated that TL and FL hydrodynamics are highly influenced by the morphological configuration of AD and that FL haemodynamics strongly depends on cumulative tear size

Marconi et al. (2017)	The authors analysed the impact of size and location of the entry tear on false lumen pressure, considered to be a dissection growth predictor, using a simplified AD phantom in a pulsatile *in vitro* circulatory loop. They found that although tears have a high impact on the coupling between TL and FL, tears alone might not be responsible for the different behaviours observed in clinical practice

Zadrazil et al. (2020)	The authors investigated the effect of the entry and re-entry tear sizes and ratio in AD on critical parameters such as flow rate, pressure and wall shear stress, using both computational and experimental models

Birjiniuk et al., 2017, Birjiniuk et al., 2020	The authors fabricated an AD silicone phantom based on clinical computed tomography (CT) images. Even though the model did not include branch vessels, it represented the first attempt to create more anatomically accurate AD phantoms. In a second work, Birjiniuk et al. studied the IF motion in phantoms perfused with pulsatile physiological flow and observed new haemodynamic features compared to the use of continuous flow, including flow reversal, large exit tear vortices and pumping action from the IF. This demonstrated the importance of employing pulsatile flow as close as possible to the physiological one

### 1.2 Objective

In Bonfanti et al. (2020) we presented a combined experimental and computational approach to investigate patient-specific AD haemodynamics informed by clinical data. A state of the art experimental platform, coupled with mathematical routines to tune its components, was employed to accurately reproduce personalised AD haemodynamic conditions, by means of a controllable pulsatile pump system, an accurate Type-B AD anatomical phantom and dynamic boundary conditions. The latter were validated against *in silico* pressure and flow rate curves, and clinical data. Selected comparisons between *in silico* and *in vitro* velocity data acquired by PIV were also made to illustrate the power of the combined approach.

Following the validation and success of our *in vitro* personalisation approach, the present work aims at analysing in-depth the experimental PIV measurements, describing in detail the experimental methods, haemodynamic parameters of interest, their significance and clinical implications. The work represents a significant advance in the state of the art of experimental AD investigations, by reporting the first, high resolution, PIV measurements of patient-specific AD blood flows.

## 2 Materials and methods

### 2.1 Experimental mock circulatory loop

**Fig. 1:**
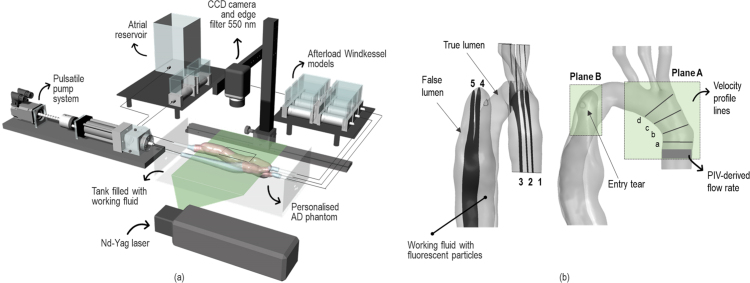
(a) Schematic illustration of the experimental platform; (b) detail of the aortic phantom showing the sections imaged with PIV (plane A1–3 and plane B4–5) as well as the location of the entry tear. The position of the four lines (a–d) where velocity profiles were extracted is also shown.

The pulsatile flow circuit comprises a computer controlled pulsatile pump and left ventricle simulator, an AD phantom, a tunable 3-element-Windkessel (3WK) model at each aortic outlet and an atrial reservoir (Fig. 1a). A detailed description of the components can be found in Franzetti et al. (2019). Pressure and flow rate waves were acquired in real time using pressure transducers (Omega Engineering, UK) and an ultrasound flow meter (Sonotec, Germany), respectively, at the inlet and outlets of the phantom. The signals were acquired via a custom made LabVIEW virtual instrument using a CompactRIO controller (cRIO-9040, 1.3 GHz Dual-Core, 70T, FPGA, RT, 4-Slot; National Instruments, USA) with a sampling sequence of 200 Hz. A Savitzky–Golay low-pass filter was employed to attenuate high frequencies in the signals.

### 2.2 Personalisation

The case study considered is based on a patient-specific dataset of a 77-year-old male subject to a chronic Type-B AD. The dataset was acquired as part of an ethical protocol (NHS Health Research Authority, ref: 12/YH/0551; Leeds Teaching Hospitals NHS Trust, ref: 788/RADRES/16), and appropriate consent was obtained from the patient. Phase-contrast through-plane velocity mapping images (2D PC-MRI) were acquired at different locations and flow information was extracted (GyroTools LCC, Zürich, Switzerland). Sphygmomanometer pressure values were also acquired. To simplify the anatomical volume of interest, the abdominal aortic branches were excluded and the domain was cut below the dissection.^1^

The experimental inlet and outlet BCs were tuned to reproduce the patient under investigation as follows. The protocol described in Franzetti et al. (2019) was adopted to obtain the analytical waveform reproducing as closely as possible the inlet aortic flow rate of the patient, measured with 2D PC-MRI. The parameters of the 3WK models were selected following the procedure described in Bonfanti et al. (2017) and Franzetti et al. (2019) with the aim of obtaining the patient-specific systolic and diastolic pressure values and correct cardiac output distribution amongst the aortic branches, shown in Table 2.

**Table 2:** Systolic ($P_{sys}$) and diastolic ($P_{dia}$) blood pressure values and mean flow rate ($\overset{¯}{Q}$) at the aortic branches used as target values for the personalisation procedure.

Parameter	Value	Unit	Source
$P_{sys}$	150	mmHg	Sphygmomanometer
$P_{dia}$	80	mmHg	Sphygmomanometer
${\overset{¯}{Q}}_{IN}$	134.5	ml/s	2D PC-MRI
${\overset{¯}{Q}}_{BT}$	22.5	ml/s	2D PC-MRI^a^
${\overset{¯}{Q}}_{LCC}$	8.9	ml/s	2D PC-MRI
${\overset{¯}{Q}}_{LSA}$	9.8	ml/s	2D PC-MRI^a^
${\overset{¯}{Q}}_{DA}$	93.3	ml/s	2D PC-MRI

a Values calculated based on patient-specific 2D PC-MRI data and physiological considerations as explained in Bonfanti et al. (2018).

### 2.3 Patient-specific phantom

The geometry of the aortic model was created with the software ScanIP (Synopsys, Mountain View, CA, USA). From CT scans, one entry tear was located approximately 10 mm distal to the proximal end of the dissection; no other communication between the TL and FL was evident from the CT data. A tapered connector with circular cross-sectional area was added upstream the aortic inlet to facilitate the connection with the experimental setup and minimise the impact of the mechanical aortic valve on the flow. The reconstructed geometry was used to 3D print a rigid AD phantom (Materialise, Belgium; TuskXC2700T material).

### 2.4 Refractive index matching

The optical properties of the AD phantom posed a significant constraint in the selection of the working fluid. The mixture was formulated in order to match the refractive index of the phantom material as closely as possible (RI=1.50). A potassium thiocyanate (KSCN) water solution (63% by weight) (RI=1.48) was selected as a Newtonian blood mimicking fluid as in previous works (Najjari et al., 2016, Gijsen et al., 1999).

Since it was not possible to create a test fluid with the same viscosity and density as blood whilst maintaining the required optical index matching with the phantom wall, a compromise had to be made in accepting a higher fluid density ($\rho =1310\;{\mathrm{kg/m}}^{3}$) and a lower dynamic viscosity (μ=2.2cP). However, the non-dimensional parameters characterising the experimental flow remained in the physiological range. Based on the aortic inlet cross-sectional area, the mean ($Re_{m}$) and peak ($Re_{p}$) Reynolds numbers^2^ were equal to 3472 and 11 581, respectively, while the Womersley number (Wo) was 32. The impact of this approximation was further investigated computationally in Bonfanti et al. (2020) by comparing the results against a non-Newtonian blood model (i.e. Carrau–Yasuda viscosity model). The working fluid approximation was deemed acceptable, as reported in Section 5.

### 2.5 PIV apparatus and acquisition protocol

PIV measurements were performed using a pulsed Nd:YAG laser (Bernoulli PIV laser B-PIV 100-25, Litron Lasers, UK) and a CCD camera (Powerview Plus 12 MP, 8 bit, Imperx, USA). Light sheet optics were used to create a laser sheet approximately 1 mm thick. The flow was seeded with Rhodamine B fluorescent polymer particles (PMMA-RhB-10, 1–20 μm; Dantec Dynamics, Denmark) and a 550 nm cutoff filter was employed. The ability of the particles to follow the flow was verified via the Stokes number (St).^3^

Image acquisition was performed using the TSI Insight4G software (TSI Inc., USA), that was also used to synchronise the camera and the laser pulses via a Laser-Pulse synchroniser (TSI Inc., USA, Model 610006). 180 pairs of particle images were recorded at a sampling rate of 22 Hz with a resolution of 4000 × 3000 pixels and a time interval between pulses of 1 ms. Given the dynamic nature of the flow field, the latter was increased to capture low flow regions (i.e. FL planes) to ensure good cross-correlation. A total of 10 cardiac cycles – and 18 instants per cycle – were acquired. Separate measurements were taken to acquire the flow field in the aortic arch – Plane A, sections 1-3 – and in the proximal part of the TL and FL in the descending aorta – Plane B, sections 4-5 – as shown in Fig. 1.

The acquired images were first processed using custom-made MATLAB functions to enhance the contrast of the image and highlight the particles, and background subtraction was performed. The images were subsequently processed using PIVlab (Thielicke and Stamhuis, 2014). Velocity fields were generated using the fast Fourier transform based cross-correlation algorithm, implemented with a three-pass technique, starting with an interrogation area of 64 × 64 pixels and ending with an area of 32 × 32 pixels, overlapping by 50%. A vector validation process was performed using the normalised median test (5 × 5) and a smoothing filter was applied to decrease the amount of noise introduced by the algorithm and increase the quality of the velocity estimation (Raffel et al., 1998).

### 2.6 PIV data analysis

Post-processing was performed to extract the parameters of interest with custom developed MATLAB (MathWorks, USA) routines.

#### Flow decomposition

The Reynolds decomposition was used to decompose a measured time-varying signal u(t)=(u,v) into a phase-averaged part 〈u(t)〉 and a fluctuating part $u^{\prime }\left(t\right)$ as:

(1) $$u\left(t\right)=\langle u\left(t\right)\rangle +u^{\prime }\left(t\right)$$

The phase-averaged term 〈u(t)〉 includes both the mean flow value and the periodic fluctuations, and was calculated as

(2) $$\langle u_{i}\rangle =\frac{1}{N}\overset{N}{\underset{n=1}{\sum }}u_{i}\;\;\;\;\forall i=1...I$$

where N is the total number of considered cardiac cycles and i denotes the instant in the cycle (I is the total number of phases, corresponding to time instances acquired in a cardiac cycle). The fluctuating component $u_{RMS}^{\prime }\left(u^{\prime },v^{\prime }\right)$ was derived as

(3) $$u_{RMS,i}^{\prime }=\sqrt{\frac{\overset{N}{\underset{n=1}{\sum }}{\left(u_{i}-\langle u_{i}\rangle \right)}^{2}}{N}}\;\forall i=1...I$$

and includes small-scale turbulent motions as well as experimental error and inter-cycle variability.

Convergence was evaluated by plotting the phase-averaged velocity in small regions as a function of the number of cycles considered; 10 cardiac cycles were found sufficient to reach converged average values to within <1.6%.

#### Turbulent kinetic energy and Reynolds shear stress

The Reynolds Shear Stress (RSS) and turbulent kinetic energy (TKE) were calculated from the fluctuating components of the velocity. These quantities characterise the level of fluctuations in the aortic flow field throughout the cardiac cycle, as well as the cycle-to-cycle variations in the fluid.

The Reynolds Stresses (i.e. $\bar{v^{\prime }v^{\prime }}$, $\bar{u^{\prime }u^{\prime }}$ and $\bar{u^{\prime }v^{\prime }}$) are obtained from the time-averaging operation over the Navier–Stokes equations and measure the momentum fluxes due to the unsteady turbulent motions. Since RSS are not invariant to coordinate rotation, principal RSS ($RSS_{max}$) were used in this work to evaluate the maximum value and avoid underestimation (Jhun et al., 2018). $RSS_{max}$ were calculated in MATLAB according to Eq. (4):

(4) $$RSS_{max}=\rho \;\sqrt{{\left(\frac{\langle u^{\prime }u^{\prime }\rangle -\langle v^{\prime }v^{\prime }\rangle }{2}\right)}^{2}+{\left(\langle u^{\prime }v^{\prime }\rangle \right)}^{2}}$$

The Turbulent Kinetic Energy (TKE) is a measure of the kinetic energy associated with turbulent eddies and indicates turbulence intensity. For a 2D flow, it is calculated as

(5) $$TKE=\frac{1}{2}\;\rho \;\left(\langle u^{\prime 2}\rangle +\langle v^{\prime 2}\rangle \right)$$

#### Vorticity and shear rate

The vorticity ω describes the local rotation of the fluid giving an indication of the presence of vortical structures in the domain. Vorticity is defined as the curl of the velocity vector 〈u〉. The component orthogonal to the plane of measurement was calculated as

(6) $$\omega =\frac{\partial v}{\partial x}-\frac{\partial u}{\partial y}$$

The shear strain rate $\overset{˙}{\gamma }$, a measure of angular deformation, was calculated as

(7) $$\overset{˙}{\gamma }=\frac{\partial u}{\partial y}+\frac{\partial v}{\partial x}$$

Both equations are numerically solved in MATLAB (Thielicke and Stamhuis, 2014) using the central difference approximation, which is a second order differentiating scheme.

#### PIV uncertainty

Several factors affect the uncertainty related to PIV measurements, which is thus inherently difficult to estimate (e.g. experimental setup alignment, timing errors, particle tracing capabilities, image quality and illumination, processing errors Sciacchitano, 2019).

A simple test to assess the overall accuracy of the PIV measurements was used in this work: the error was quantified by estimating the mass imbalance (Sherwood et al., 2014).^4^ The difference between the flow rate values led to a mass imbalance error of 5.32%, which was deemed acceptable.

## 3 Results and discussions

### 3.1 Personalisation procedure

As a result of the personalisation procedure, the experimental inlet flow rate curve had a total cycle time of 0.82 s, resulting in a heart frequency of 73 bpm, mean flow rate of 137.41 ml/s and SV of 106 ml, closely matching the parameters extracted from the 2D PC-MRI.

**Fig. 2:**
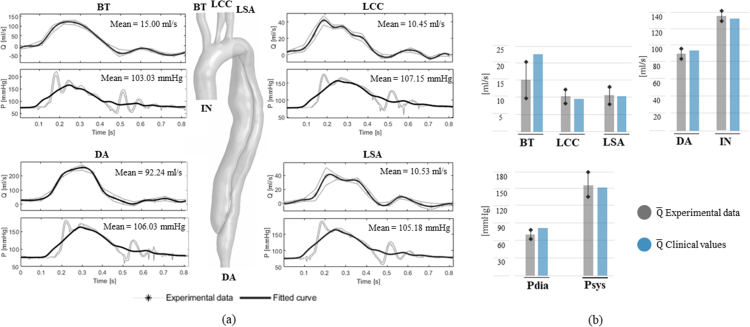
(a) Experimental flow rate and pressure curves, and their mean value, acquired at the outlets of the anatomical domain. The oscillations observed in the pressure curves are due to the action of the mechanical valve and correspond to its opening and closing phases. (b) Comparison between experimental mean flow rate values at the aortic outlets and systolic ($P_{sys}$) and diastolic ($P_{dia}$) pressures at the inlet and target clinical values. Error bars are reported for the experimental data, which include the standard deviation (SD) due to inter-cycle variability as well as the amplitude of the experimental oscillations due to the mechanical action of the aortic valve for the systolic pressure value shown in the histogram.

The physical 3WK models successfully reproduced the hydraulic impedance of the distal vasculature of the aorta, allowing to reproduce physiological pressure/flow relationships, as evident from the realistic waveforms acquired in the experiment as shown in Fig. 2a. The data was compared to the target clinical information via histograms in Fig. 2b. The clinical systolic/diastolic pressure values and mean flow rate at the outlets are within the experimental range with the only exception of BT, where the clinical value exceeds the upper experimental bound by 1.5 ml/s, equivalent to an error of 6%.

By tuning the resistance and compliance of each 3WK (Table 3), it was possible to personalise important haemodynamic quantities, such as the cardiac output distribution among the aortic branches and the systolic/diastolic pressure values.

**Fig. 3:**
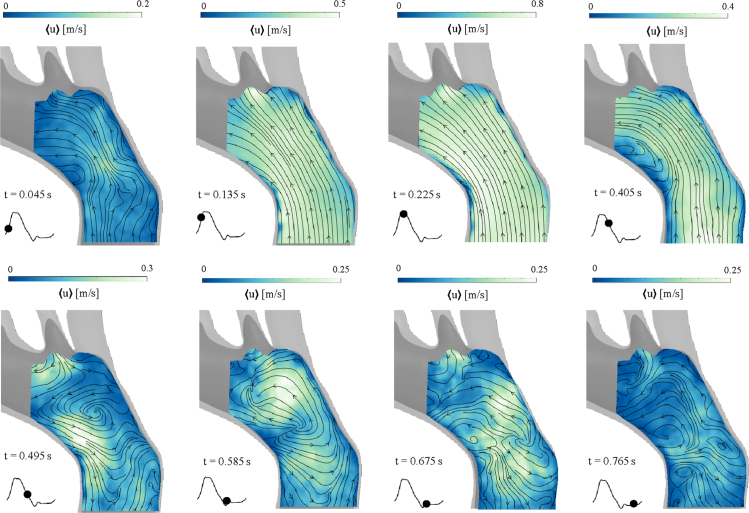
Phase-averaged velocity magnitude fields 〈u〉 in the proximal part of the aortic arch (Plane A2) at eight instants of the cardiac cycle. Contours represent the velocity magnitude and overlapped streamlines indicate the flow direction. The figure shows how the flow develops over time, changing from organised flow in systole to complex flow patterns and flow reversals in diastole. Different colour contour ranges were used to highlight the flow features.(For interpretation of the references to colour in this figure legend, the reader is referred to the web version of this article.)

**Fig. 4:**
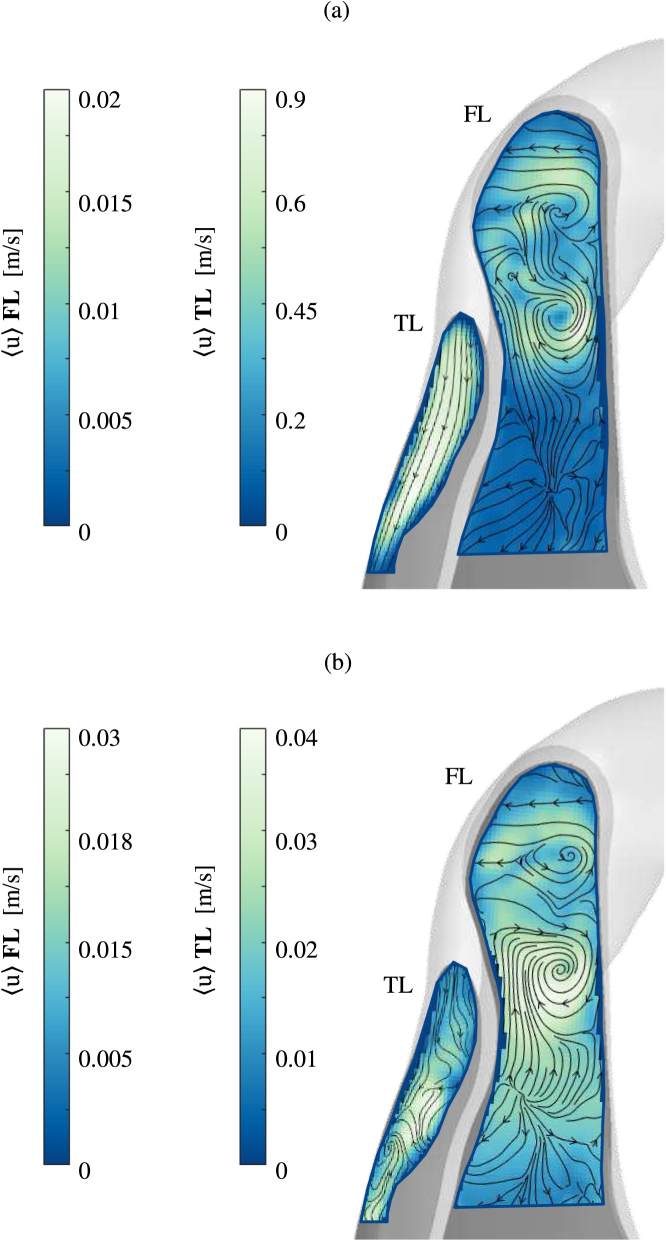
Phase-averaged velocity magnitude fields 〈u〉 in the proximal part of TL and FL (Plane B4) at (a) peak systole, (b) diastole. Contours represent the velocity magnitude and overlapped streamlines indicate the flow direction. Different contours levels had to be selected due to the difference in velocity values, particularly during systole.

**Fig. 5:**
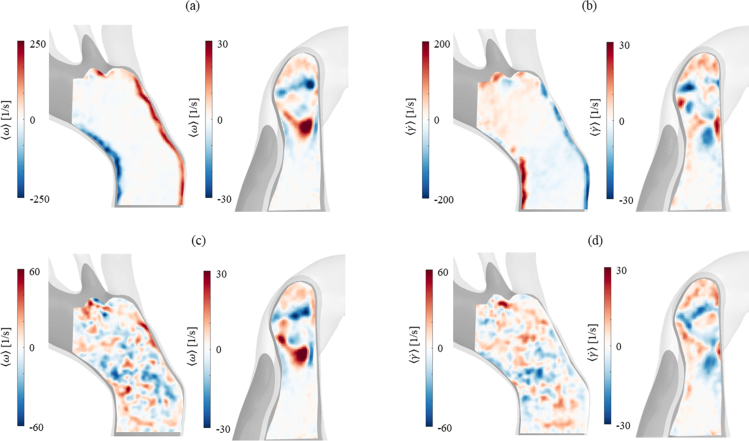
Vorticity (ω) and shear rate $\left(\overset{˙}{\gamma }\right)$ contour plots in the aortic arch and proximal part of the FL at (a,b) peak systole and (c,d) diastole. It should be noted that the vorticity observed in the aortic arch at the boundary walls is not related to the formation of vortical structures but induced by shear. Different contours levels had to be selected to show flow features because of the difference in values between systole and diastole.

**Fig. 6:**
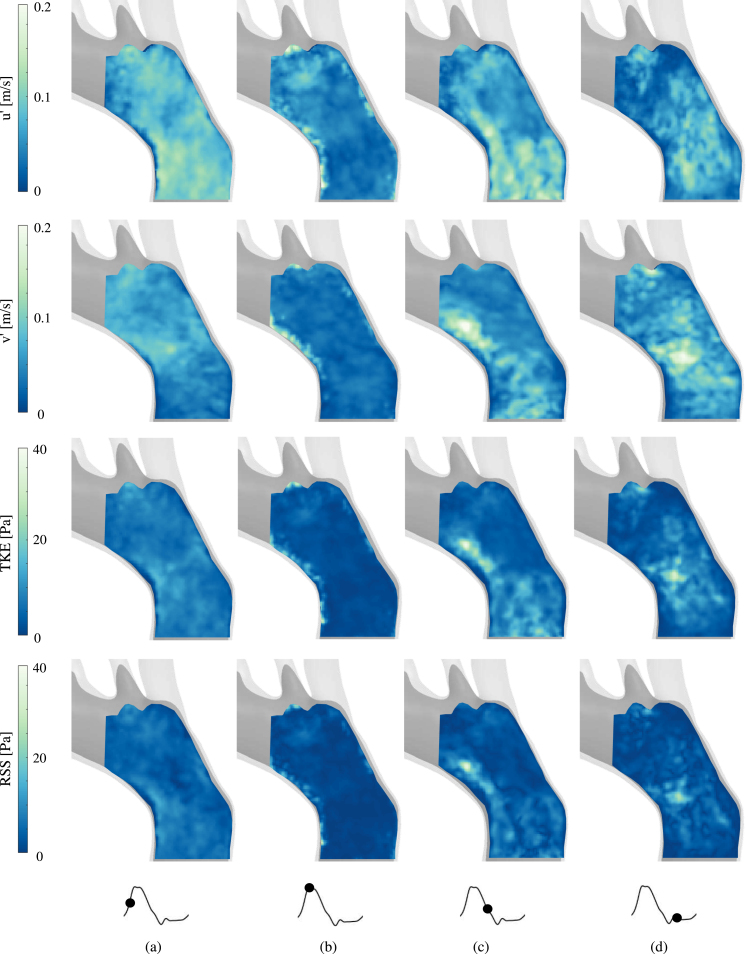
Contour plots of $u^{\prime }$, $v^{\prime }$, TKE and RSS in the proximal part of the aortic arch (Plane A2) at (a) acceleration, (b) peak systole, (c) deceleration and (d) diastole.

**Table 3:** Values of the resistances ($R_{1}$ and $R_{2}$) and compliance (C) of the 3WK of the branches of the 0D model for the patient.

3WK	$R_{1}$	$R_{2}$	C
	mmHg s ml${}^{-1}$	mmHg s ml${}^{-1}$	ml mmHg${}^{-1}$
BT	0.25	6.00	0.30
LCC	0.63	10.00	0.07
LSA	0.57	10.00	0.07
DA	0.01	1.15	0.30

### 3.2 Evolution of flow patterns

PIV-derived, phase-averaged velocity magnitude distributions in the proximal part of the aortic arch are illustrated in Fig. 3.^5^ Highly organised motion with parallel streamlines is observed during the systolic phase, reaching a velocity of 0.8 m/s^6^ at peak systole (t = 0.225 s). A flow separation region can be observed at the beginning of deceleration – at early diastole (t = 0.405 s) – characterised by flow re-circulation and low magnitude of flow velocity, with retrograde flow along the inner wall and antegrade flow along the outer wall of the aortic arch. Disorganised and complex flow fields, arising from flow reversal, characterise the diastolic phase, with velocities varying throughout the domain and reaching up to 0.25 m/s.

Fig. 4 shows the phase-averaged velocity field in the proximal part of the descending aorta, where both TL and FL are present, at peak systole and diastole. The TL exhibits the same behaviour observed in the aortic arch; at peak systole the flow is well organised while during diastole non-organised streamlines characterise the flow behaviour. Higher velocities are observed in this region in the TL, with maximum values reaching 0.9 m/s due to the dissection causing a narrowing to the cross-sectional area (Fig. 4a, TL). Due to the absence of a secondary tear in the IF, and rigid wall approximation, there is not significant blood flow in the FL and measured velocities are low, with values <0.05 m/s. Recirculation regions, stagnant and disorganised flow are observed in this area during the whole cardiac cycle. In particular, two main counter rotating vortices can be noted, due to the separation of the flow entering the FL, as indicated by the streamlines (Fig. 4). This is a common feature observed in AD flow, as reported in computational studies (Cheng et al., 2014). Unlike the TL, no significant changes in the velocity magnitude or flow behaviour are observed in the FL during the cardiac cycle. Higher velocity values are captured in the FL during diastole because of the particular location of the imaged plane (see Fig. 1b), which does not include the entry tear and is oriented perpendicularly to its cross section. During systole, the flow is mainly out of the imaged plane, and hence the measured velocities are lower, whereas during diastole in-plane recirculating flows develop therefore increasing the measured velocities.

### 3.3 Haemodynamic indices

The evolution of vorticity during the cardiac cycle, in both the TL and FL, is shown in Fig. 5. Viscosity-induced friction shear at the wall, which acts as a source of vorticity, is evident in near-wall regions in the ascending part of the aortic arch during the cardiac cycle. In the aortic arch and TL vorticity varies in time, highlighting the three-dimensional and disorganised flow patterns during deceleration and diastole, whilst in systole (Fig. 5a, aortic arch) an irrotational flow region is observed outside the near-wall boundaries. On the contrary, vortex formation is present throughout the cardiac cycle in the FL and the two vortices noticed in the velocity field (Fig. 4) are here evident; one clockwise and one anticlockwise, with vorticity up to 30 s${}^{-1}$, both in the proximity of the tear location (shown in Fig. 1).

Low values of shear rate are found in the FL, as shown in Fig. 5b,d. This behaviour, together with small vortices and stagnant flow, has been linked to FL thrombus formation in a murine model of AD (Yazdani et al., 2018), where low shear rate was found at both ends of the FL, regions in which thrombus starts forming. In the same area, clot formation was noticed in the clinical images of the patient under investigation as well.

In Fig. 6, $u^{\prime }$, $v^{\prime }$, TKE and RSS distributions in the proximal part of the aortic arch (Plane A2) are shown. The fluctuating velocity varies in magnitude over the cardiac cycle. During systole (Fig. 6b), when the flow exhibits little to no disruption and the fluid flows in parallel layers (as shown in Fig. 3), the fluctuating velocity is negligible compared to the mean flow velocity. These fluctuations represent the expected cycle-to-cycle variation and experimental error, in particular close to the aortic wall. During acceleration, deceleration and diastole (Fig. 6a,c,d) – the flow fields become disorganised and flow reversal takes place enhancing flow mixing (as shown in Fig. 3) – the fluctuating velocity exhibits a more dominant behaviour, with higher values. The magnitude of the fluctuating velocities becomes now comparable with the phase-averaged one.

Similarly TKE and $RSS_{max}$ show higher values during deceleration and diastole. TKE shows low values during acceleration and peak systole due to the stabilising effect of the flow acceleration (Fig. 6a,b). Then, it reaches a maximum value of 40 Pa (0.03 $m^{2}\;s^{-2}$) during deceleration and diastole (Fig. 6c,d) – where higher $u^{\prime }$, $v^{\prime }$ values are observed – and decreases reaching its minimum value during acceleration in the next cardiac pulse. The observed evolution of TKE over the cardiac cycle is in agreement with the one reported by Kousera et al. (2012) in a rigid CFD model in the ascending aorta.

## 4 Clinical implications

The characterisation of local haemodynamics, through indices like the ones shown in this work, can serve as a tool for disease prognosis and clinical intervention planning (Bonfanti et al., 2017). Haemodynamic indices such as TKE, RSS and shear rate have been associated with platelet activation, haemolysis and blood coagulation (Hatoum et al., 2018, Jhun et al., 2018, Yazdani et al., 2018), which is of clinical relevance for the FL. Indeed, whereas the complete FL thrombosis has a positive prognostic value, the absence of or partial thrombosis is a predictor of aortic dilation and death (Tsai et al., 2007). As shown by recent literature, haemodynamic markers have significant impact in the long-term progression of AD and informed clinical planning should not only rely on geometrical information but rather consider the whole complex pathological fluid dynamic environment into consideration (Bonfanti et al., 2019).

The possibility of accurately reproducing and studying personalised vascular haemodynamics *in vitro* – via experimental platforms like the one developed in this work – enables the testing of medical devices and procedures in clinically relevant settings. This can provide valuable information to support the design of new technology, as recently shown in an *in vitro* study on endograft configuration for Type-B AD (Birjiniuk et al., 2020). Realistic phantoms and blood fluid dynamics can also be employed for surgical training and pre-procedural planning of complex cases of AD.

Furthermore, the experimental approach presented in this work can provide a valuable benchmark for CFD model validation through controlled conditions and high definition fluid dynamic visualisation and measurements. Once fully validated, computational haemodynamic models can be used to estimate with high detail markers that would be difficult to measure experimentally and impossible *in vivo* (e.g. WSS) and to virtually simulate different surgical interventions (e.g. fenestration or tear occlusion). The personalisation showcased in this work represents a leap forward in experimental fluid dynamic modelling, further narrowing the gap between the latter and state of the art numerical simulations, hence allowing their extensive validation (Bonfanti et al., 2020).

## 5 Limitations

This first attempt to experimentally characterise patient-specific AD flow is subject to some limitations.

Firstly, a compromise had to be made with regards to the rheology of the blood mimicking fluid. However, the impact of this approximation was evaluated numerically by comparing the results against a simulation run with rheological properties of a non-Newtonian blood model,^7^ and was deemed acceptable (Bonfanti et al., 2020).^8^ Nonetheless, non-Newtonian behaviour can play a significant role in AD and, in particular in the FL, and future work will therefore address this limitation.

The assumption of rigid wall of the aortic phantom represents the second main limitation of this work. The difference between the rigid wall model and the compliant one was described in Bonfanti et al. (2018) for the same case-study, highlighting a maximum wall displacement of less that 0.75 mm in the arch and descending aorta. Consequently, the rigid wall motion was deemed acceptable for the purpose of this study. Nonetheless, this approximation is not always appropriate and the manufacturing of compliant phantoms will be addressed in future work.

Lastly, only two velocity components were acquired using a 2D PIV system, even though the flow presents 3D features, and the low temporal-resolution represents an issue for turbulence characterisation, affecting the accuracy of the estimation of haemodynamic indices such as TKE and RSS. However, when the flow under investigation has an axial velocity greater than the other two components, the analysis of the 2D data can be regarded as a good approximation of the 3D counterpart (Barbaro et al., 1998). Nevertheless, reconstructing the flow from parallel measured planes is fraught with difficulties in the case of this geometry, hence future work with time-resolved volumetric or tomographic PIV is planned to resolve the 3D nature of the flow and improve turbulent statistics.

## 6 Conclusions

The work described presents the first experimental patient-specific model of AD and represents a significant advancement of the state of the art of *in vitro* AD haemodynamic modelling. The entirety of published *in vitro* models of AD are limited by simplified assumptions regarding the choice of BCs as well as, often, of the morphological features of the vessel.

In contrast, in this work, both vessel geometry and dynamic, physiological BCs were accounted for, tuned by clinical data to enable the flow platform to reproduce *personalised* patient conditions. The experimental flow and pressure waveforms obtained were validated against *in vivo* data and demonstrated the capability of the tuning procedures. Moreover, for the first time, clinically relevant haemodynamic parameters were extracted *in vitro* in a patient-specific model from PIV data. These parameters compared well with both the known physiological behaviour and literature findings, and showcase the potential of performing such complex experimental AD studies.

The ability to reproduce accurate, personalised AD cases *in vitro*, and extract relevant information can be used not only to obtain insights into the pathology of the disease but also provides a physical test benchmark and a validation tool for the clinical translation of state of the art numerical AD models. With the ultimate goal of working towards *precision medicine* – or *patient stratified medicine* – the methodologies and devices presented in this work have the potential to facilitate clinical translation of experimental as well as numerical tools.

## CRediT authorship contribution statement

**Gaia Franzetti:** Conceptualisation, Formal analysis, Investigation, Methodology, Visualisation, Writing – original draft. **Mirko Bonfanti:** Conceptualisation, Methodology, Software, Writing – review & editing. **Shervanthi Homer-Vanniasinkam:** Conceptualisation, Resources. **Vanessa Diaz-Zuccarini:** Conceptualisation, Funding acquisition, Project administration, Supervision. **Stavroula Balabani:** Conceptualisation, Funding acquisition, Project administration, Supervision, Writing – review & editing.

## Declaration of Competing Interest

The authors declare that they have no known competing financial interests or personal relationships that could have appeared to influence the work reported in this paper.

## Footnotes

1 The resulting geometry included the brachiocephalic trunk (BT), left common carotid (LCC), left subclavian artery (LSA), and descending aorta (DA).

2 Since $Re_{p}$ was higher than the critical Reynolds number ($Re_{c}$) for transition to turbulent flow (calculated following Kousera et al. (2012) as $Re_{c}=250\times Wo$), the flow is expected to be turbulent.

3 Defined as $St=\tau_{p}\cdot u/D_{l}$, where $\tau_{p}$ is the characteristic time of the particles calculated as $\tau_{p}=D_{p}^{2}\rho_{p}/18\mu$. $D_{l}$ is the minimum aortic true lumen diameter, u is the peak velocity, $D_{p}$ is the mean particle diameter, $\rho_{p}$ is the particle density and μ is the fluid dynamic viscosity. The estimated value was much lower than unity (St≪1), meaning that the particles faithfully followed the flow velocity.

4 Assuming a constant density and rigid walls, conservation of mass requires that the volumetric flow rate is constant along the considered section. The flow rate was estimated from the phase-averaged PIV velocity field performing a double integral under the assumption of semi-axisymmetric flow, at the inlet of the aorta (i.e. rigid cylinder with a circular cross-sectional area).

5 Results obtained from all planes (i.e. Plane A1–3 and B4–5, as shown in Fig. 1b) were analysed and the same velocity ranges and distributions were observed. Planes A2 and B4 were selected as representative as the ones closer to the vessel centre-line, affected less by wall refraction.

6 The average aortic inlet velocity was 0.18 m/s while the maximum inlet velocity, at peak systole, was 0.6 m/s.

7 Carreau–Yasuda viscosity model with parameters from Clarion et al. (2018) and ρ= 1060 kg/m${}^{3}$.

8 A maximum and mean difference of 25.9 and 1.0%, respectively, was found when comparing the velocity fields at peak systole in the whole fluid domain, while a maximum difference of 3.6 and 4.3% was found between the P and Q waveforms at the boundaries.

## References

[b1] Barbaro V., Grigioni M., Daniele C., D’Avenio G. (1998). Principal stress analysis in LDA measurements of the flow field downstream of 19-mm Sorin Bicarbon heart valve. Technol. Health Care.

[b2] Birjiniuk J., Oshinski J.N., Ku D.N., Veeraswamy R.K. (2020). Endograft exclusion of the false lumen restores local hemodynamics in a model of type B aortic dissection. J. Vasc. Surg..

[b3] Birjiniuk J., Timmins L.H., Young M., Leshnower B.G., Oshinski J.N., Ku D.N., Veeraswamy R.K. (2017). Pulsatile flow leads to intimal flap motion and flow reversal in an in vitro model of type b aortic dissection. Cardiovasc. Eng. Technol..

[b4] Bonfanti M., Balabani S., Alimohammadi M., Agu O., Homer-vanniasinkam S., Díaz-zuccarini V. (2018). A simplified method to account for wall motion in patient-specific blood flow simulations of aortic dissection : Comparison with fluid-structure interaction. Med. Eng. Phys..

[b5] Bonfanti M., Balabani S., Greenwood J.P., Puppala S., Homer-Vanniasinkam S., Díaz-Zuccarini V. (2017). Computational tools for clinical support: a multi-scale compliant model for haemodynamic simulations in an aortic dissection based on multi-modal imaging data. J. R. Soc. Interface.

[b6] Bonfanti M., Franzetti G., Homer-Vanniasinkam S., Diaz-Zuccarini V., Balabani S. (2020). A combined in vivo, in vitro, in silico approach to study the patient-specific haemodynamics of Type-B Aortic Dissections. Ann. Biomed. Eng..

[b7] Bonfanti M., Franzetti G., Maritati G., Homer-Vanniasinkam S., Balabani S., Diaz-Zuccarini V. (2019). Patient-specific haemodynamic simulations of complex aortic dissections informed by commonly available clinical datasets. Med. Eng. Phys..

[b8] Cheng Z., Wood N.B., Gibbs R.G.J., Xu X.Y. (2014). Geometric and flow features of type b aortic dissection: Initial findings and comparison of medically treated and stented cases. Ann. Biomed. Eng..

[b9] Chung J.W., Elkins C., Sakai T., Kato N., Vestring T., Semba C.P., Slonim S.M., Dake M.D. (2000). True-lumen collapse in aortic dissection: part I. Evaluation of causative factors in phantoms with pulsatile flow. Radiology.

[b10] Chung J.W., Elkins C., Sakai T., Kato N., Vestring T., Semba C.P., Slonim S.M., Dake M.D. (2000). True-lumen collapse in aortic dissection: part II. Evaluation of treatment methods in phantoms with pulsatile flow. Radiology.

[b11] Clarion M., Deegan M., Helton T., Hudgins J., Monteferrante N., Ousley E., Armstrong M. (2018). Contemporary modeling and analysis of steady state and transient human blood rheology. Rheol. Acta.

[b12] Dziodzio T., Juraszek A., Reineke D., Jenni H., Zermatten E., Zimpfer D., Stoiber M., Scheikl V., Schima H., Grimm M., Czerny M. (2011). Experimental acute type b aortic dissection: Different sites of primary entry tears cause different ways of propagation. ATS.

[b13] Faure E.M., Canaud L., Cathala P., Serres I., Marty-Ané C., Alric P. (2014). Human ex-vivo model of stanford type B aortic dissection. J. Vasc. Surg..

[b14] Franzetti G., Diaz-Zuccarini V., Balabani S. (2019). Design of an in vitro mock circulatory loop to reproduce patient-specific vascular conditions: towards precision medicine. ASME JESMDT.

[b15] Gijsen F.J.H., Vosse F.N.V.D., Janssen J.D. (1999). The influence of the non-Newtonian properties of blood on the flow in large arteries: steady flow in a carotid bifurcation model. J. Biomech..

[b16] Girish A., Padala M., Kalra K., Mciver B.V., Veeraswamy R.K., Chen E.P., Leshnower B.G. (2016). The impact of intimal tear location and partial false lumen thrombosis in acute type B aortic dissection. Ann. Thorac. Surg..

[b17] Hatoum H., Yousefi A., Lilly S., Maureira P., Crestanello J., Dasi L.P. (2018). An in vitro evaluation of turbulence after transcatheter aortic valve implantation. J. Thorac. Cardiovasc. Surg..

[b18] Jhun C.S., Stauffer M.A., Reibson J.D., Yeager E.E., Newswanger R.K., Taylor J.O., Manning K.B., Weiss W.J., Rosenberg G. (2018). Determination of Reynolds shear stress level for hemolysis. ASAIO J..

[b19] Kousera C.A., Wood N.B., Seed W.A., Torii R., O’Regan D., Xu X.Y. (2012). A numerical study of aortic flow stability and comparison with in vivo flow measurements. J. Biomech. Eng..

[b20] Marconi C.A. (2017). A novel insight into the role of entry tears in type B aortic dissection: pressure measurements in an in vitro model. Int. J. Artif. Organs..

[b21] Morales D.L.S., Quin J.A., Braxton J.H., Hammond G.L., Gusberg R.J., Elefteriades J.A. (1998). Experimental confirmation of effectiveness of fenestration in acute aortic dissection. Ann. Thorac. Surg..

[b22] Najjari M.R., Hinke J.A., Bulusu K.V., Plesniak M.W. (2016). On the rheology of refractive-index-matched, non-Newtonian blood-analog fluids for PIV experiments. Exp. Fluids.

[b23] Peelukhana S.V., Wang Y., Berwick Z., Kratzberg J., Krieger J., Roeder B., Cloughs R.E., Hsiao A., Chambers S., Kassab G.S. (2016). Role of pulse pressure and geometry of primary entry tear in acute type B dissection propagation. Ann. Biomed. Eng..

[b24] Raffel M., Willert C., Wereley S., Kompenhans J. (1998).

[b25] Rudenick P.A., Bijnens B.H., García-Dorado D., Evangelista A. (2013). An in vitro phantom study on the influence of tear size and configuration on the hemodynamics of the lumina in chronic type B aortic dissections. J. Vasc. Surg..

[b26] Sciacchitano A. (2019). Uncertainty quantification in particle image velocimetry. Meas. Sci. Technol..

[b27] Sherwood J.M., Holmes D., Kaliviotis E., Balabani S. (2014). Spatial distributions of red blood cells significantly alter local haemodynamics. PLoS One.

[b28] Thielicke W., Stamhuis E.J. (2014). Pivlab – towards user-friendly, affordable and accurate digital particle image velocimetry in MATLAB. J. Open Res. Softw..

[b29] Tsai T.T., Evangelista A., Nienaber C.A., Myrmel T., Meinhardt G., Cooper J.V., Smith D.E., Suzuki T., Fattori R., Llovet A., Froehlich J., Hutchison S., Distante A., Sundt T., Beckman J., Januzzi J.L., Isselbacher E.M., Eagle K.A. (2007). Partial thrombosis of the false lumen in patients with acute type B aortic dissection for the international registry of acute aortic dissection. N. Engl. J. Med..

[b30] Van Bogerijen G.H., Tolenaar J.L., Rampoldi V., Moll F.L., Van Herwaarden J.A., Jonker F.H., Eagle K.A., Trimarchi S. (2014). Predictors of aortic growth in uncomplicated type B aortic dissection. J. Vasc. Surg..

[b31] Yazdani A., Li H., Bersi M.R., Di Achille P., Insley J., Humphrey J.D., Karniadakis G.E. (2018). Data-driven modeling of hemodynamics and its role on thrombus size and shape in aortic dissections. Sci. Rep..

[b32] Yuan X., Clough R.E., Nienaber C.A. (2020). Management of uncomplicated type B aortic dissection. Hearts.

[b33] Zadrazil I., Corzo C., Voulgaropoulos V., Markides C.N., Xu X.Y. (2020). A combined experimental and computational study of the flow characteristics in a type b aortic dissection: Effect of primary and secondary tear size. Chem. Eng. Res. Des..

